# Field Test of the MiniRadMeter Gamma and Neutron Detector for the EU Project CLEANDEM

**DOI:** 10.3390/s24185905

**Published:** 2024-09-11

**Authors:** Marco Ripani, Fabio Rossi, Luigi Cosentino, Fabio Longhitano, Paolo Musico, Mikhail Osipenko, Gaetano Elio Poma, Paolo Finocchiaro

**Affiliations:** 1 INFN Sezione di Genova, 16146 Genova, Italy; marco.ripani@ge.infn.it (M.R.); fabio.rossi@ge.infn.it (F.R.); paolo.musico@ge.infn.it (P.M.); mikhail.osipenko@ge.infn.it (M.O.); 2 INFN Laboratori Nazionali del Sud, 95123 Catania, Italy; cosentino@lns.infn.it (L.C.); elio.poma@lns.infn.it (G.E.P.); 3 INFN Sezione di Catania, 95123 Catania, Italy; fabio.longhitano@ct.infn.it

**Keywords:** gamma detector, neutron detector, nuclear decommissioning, nuclear accident remediation, robotic radiological inspection

## Abstract

In the framework of the H2020 CLEANDEM project, a small robotic vehicle was equipped with a series of different sensors that were developed for the preliminary inspection of areas possibly contaminated by radiation. Such unmanned inspection allows to identify dangerous locations prior to the possible start of human operations. One of the developed devices, named the MiniRadMeter, is a compact low-cost sensor that performs gamma and neutron radiation field mapping in the environment. The MiniRadMeter was successfully tested in a simulated field mission with four “hidden” radioactive sources and a neutron generator. In this work, we describe the test procedure and the results, which were supported by the outcome of dedicated Monte Carlo simulations.

## 1. Introduction

**Figure 1:**
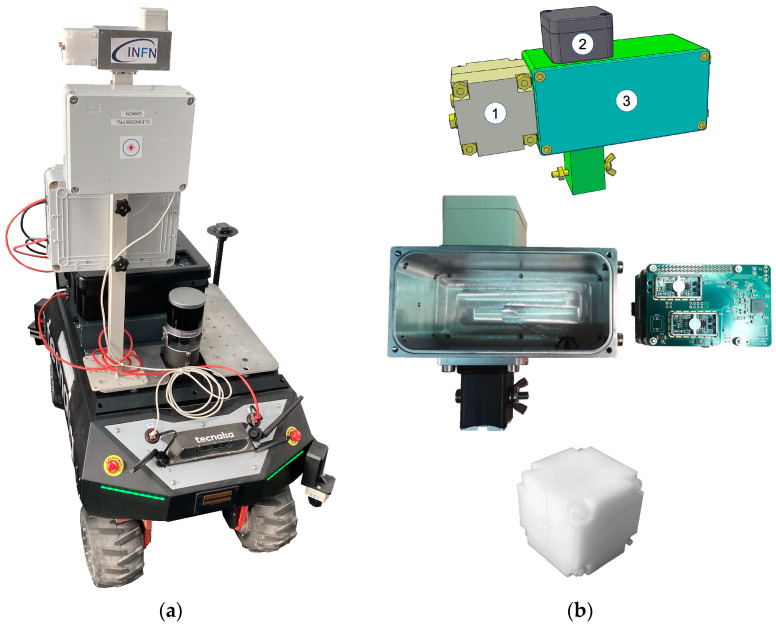
(**a**) The CLEANDEM UGV. (**b**) The MiniRadMeter device hosting the neutron moderator and detector (1); the gamma detector (2); the front-end electronics and data acquisition microcomputer (3).

The present and future nuclear decommissioning activities, along with possible accident remediations, will certainly require less physical presence of human operators in potentially radiologically hostile environments and the consequent risks by means of robotic equipment. The CLEANDEM project (Cyber physicaL Equipment for unmAnned Nuclear DEcommissioning Measurements), whose motto is “Safer, Cleaner, Better, Faster, Cheaper”, was funded by the framework of the EU Horizon 2020 plan [1] and was recently concluded by the construction of the foreseen UGV (unmanned ground vehicle) and its test in a realistic environment. This UGV demonstrator, perhaps the first of its kind, hosts several sensors and devices (Figure 1a) and can also be equipped with a special arm to manipulate additional sensors. An onboard LIDAR (laser imaging detection and ranging) system provides the capability of 3D mapping of the surrounding environment, which can be associated with the radiological measurement results. An internet connection is used to provide the universal time stamp. One of the devices permanently installed on top of the UGV front pole is the MiniRadMeter, devoted to measurements of the gamma and neutron radiation in the environment and whose full description of the components and expected performances are given in [2]. It features two different sensors, a scintillator for gamma rays and a solid state diode for neutrons, both handled by compact electronics driven by a commercial microcomputer. It is well known that the main types of radioactivity are alpha, beta, gamma, neutron and fission. While alpha particles and fission fragments are immediately stopped in very thin layers of matter, with beta electrons traveling a little more but stopped, e.g., in a thin aluminum sheet, neutrons and gamma rays are much more penetrating and may require thick lead, concrete and/or water to be stopped. As a side effect, they are also easier to detect, and therefore a quick and effective radiological survey should primarily be based on gammas and neutrons, considering also that almost any kind of radioactive decay usually implies the emission of gamma radiation, while neutrons indicate the presence of alpha-emitting actinides or fissile species. Several developments of small scintillation detectors for gamma rays [3,4,5,6,7], along with compact neutron detectors [8,9], have been pursued and others are currently under way.

In this work we describe the test of the MiniRadMeter in a simulated field mission with four “hidden” radioactive sources and a neutron generator, along with the test procedure and the results, also supported by the outcome of dedicated simulations. The mission was performed on the premises of AiNT GmbH [10], a member of the CLEANDEM consortium. The full description of the UGV and all its sensors is foreseen as the subject of forthcoming papers.

## 2. Materials and Methods

### 2.1. The MiniRadMeter Sensors and the Control System

The gamma ray detector is a CsI(Tl) scintillating crystal produced by Hilger Crystals [11], 1 × 1 × 1 cm^3^ size, optically coupled to a Silicon Photo Multiplier (SiPM) MicroFC-60035-SMT [12]. It is a radiation sensor that provides good detection efficiency and medium energy resolution at a very reasonable cost below EUR 100 [2,13,14]. Upon the interaction of a gamma ray with the crystal, a short flash of light is produced, with the spectrum centered around 550 nm, a decay time of about 1 µs and a yield of 60,000 photons per deposited MeV. The scintillation light is collected by the SiPM that is a solid state photo multiplier with a photon detection efficiency (PDE) of the order of 20% at such a wavelength. Each photon interacting with the SiPM gives rise to an avalanche of about 10^6^ electrons (i.e., the SiPM has a gain of ≈10^6^) so that the resulting output signal is macroscopic. This signal only requires a slight amplification by a simple home-developed circuit [2] to match the input characteristics of the ADC featured by the Raspberry Pi4 microcomputer board [15], whose cost was around EUR 100, which drives the MiniRadMeter. The ADC samples the signal at 10 Msamples/s and produces a digital reconstruction of the input waveform. On the occurrence of a software threshold crossing, a suitable algorithm calculates the pulse area, obtaining a number that is proportional to the energy deposited by the detected gamma ray. This procedure is performed event by event and a histogram is produced. By means of a proper calibration, the detector thus provides the spectrum of the energy deposited by gamma rays, whose integral is the overall number of counts that can be quickly rescaled to dose rate dividing by the measuring time and multiplying by a suitable conversion coefficient. 

As for the neutron detector, SiLiF technology was chosen, where a film of ^6^LiF neutron converter is placed in front of a silicon detector that provides a current signal when hit by either a triton or an alpha particle emitted following a neutron capture in ^6^Li [16,17,18,19,20,21]. The detector, which requires a once-only calibration, provides the spectrum of the energy deposited by the charged particles. A suitable threshold of 1.5 MeV completely removes any significant gamma contribution, so that every count above the threshold is to be ascribed to neutrons. The chosen configuration employs two commercial silicon photodiodes produced by Hamamatsu, each one costing less than EUR 100 [22], 1 × 1 cm^2^ size, installed back-to-back, connected in parallel and each one facing a homemade ^6^LiF film deposited on a glass substrate by means of a simple chemical procedure [23]. The neutron detector assembly, whose thermal neutron detection efficiency is 5%, is placed inside a polyethylene box measuring 6 × 6 × 6 cm^3^ acting as a moderator. The moderator size was chosen after several Geant4 [24] simulations as a trade-off between the capability of moderation of high-energy neutrons and the mechanical size and weight. Indeed, the simulations showed that, in the chosen geometry the detection efficiency decreases gently by factor ten for neutron energy from thermal, 0.025 eV, up to 1 MeV compared with factor ≈ 4000 for an unmoderated detector [2]. The signal produced by the sensor is fed to a home-developed charge preamplifier [2], whose output is sampled by the second channel of the ADC hosted on the Raspberry. A mathematical transform changes the typical exponential shape of the preamplifier output into a trapezoidal one, whose flat-top height is proportional to the energy deposited into the silicon [25].

**Figure 2:**
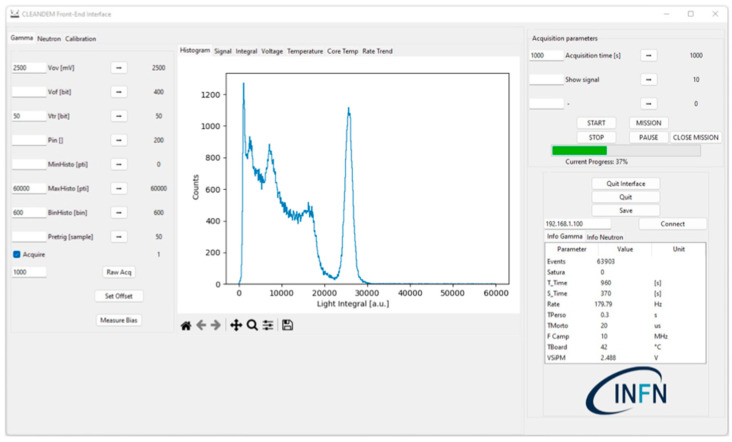
A snapshot of the user interface control screen.

Therefore, the data acquisition system has two analog front ends, one for each sensor, and a single ADC with two differential input channels handled by the Raspberry that collects, elaborates and stores the data. The microcomputer also takes care of connecting to the global storage database and optionally to the user interface software, written in Python v3.10.10 and running on a standard PC, through the UGV wireless connection or directly via its own WiFi interface. A closer view of the MiniRadMeter and its components is shown in Figure 1b. A snapshot of the user interface control screen is shown in Figure 2. 

### 2.2. Gamma Detector Calibration

**Figure 3:**
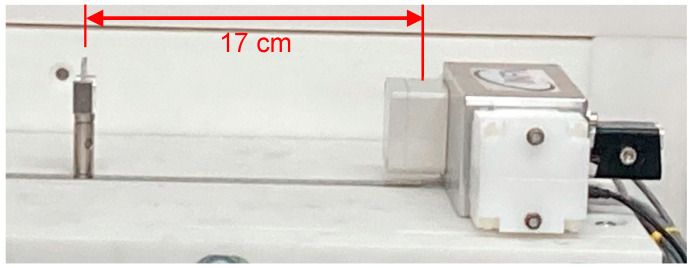
The gamma detector calibration setup with the source holder at 17 cm distance.

**Figure 4:**
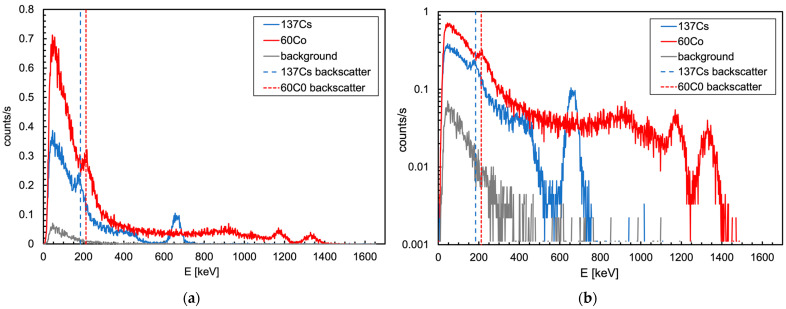
(**a**) Spectra of the ^137^Cs, ^60^Co and background in linear y scale. The Compton backscattering peaks, located exactly where expected, testify the goodness of the linear calibration. (**b**) Same plot in logarithmic y scale. The energy resolution at the ^137^Cs peak (662 keV) was 6.8%, and at the higher ^60^Co peak (1330 keV) it was 4.3%.

A preliminary calibration of the gamma detector was performed by means of two point-like sources, namely ^137^Cs (282 kBq) and ^60^Co (352 kBq). Each source was placed on a holder 17 cm from the detector (Figure 3) and a spectrum was acquired. The three energy peaks from the sources, at 662, 1173 and 1330 keV, respectively, were used to perform a linear calibration. As a cross check of the calibration goodness, we looked at the position of the 184 and 212 keV Compton backscattering peaks in the spectra, which resulted exactly where expected. The background spectrum was also acquired (Figure 4).

The count rate to dose rate conversion was performed, as indicated in [2], by referring to the usual calibration of active dosimeters based on the 662 keV gamma radiation from ^137^Cs. The equivalent dose rate produced by such a source of 1 MBq activity at 1 m distance was 0.1 µSv/h. The interaction probability in CsI(Tl) was about 30% [26], and the subtended solid angle gave rise to a flux of about 8 gamma/cm^2^/s, resulting in about 2.7 counts/s in the detector, as also verified experimentally. These considerations led to a conversion factor of about 0.037 (µSv/h)/(counts/s). In Section 4, a more precise energy-dependent conversion algorithm will be discussed.

## 3. Results: Field Mission

A simulated field mission was arranged in order to verify the behavior of the MiniRadMeter in a realistic environment. A shielded hall was made available at AiNT, which was perfect for a gamma test mission. Unfortunately, a realistic neutron detection mission was unfeasible as it would require placing an intense neutron source that (i) was not available and (ii) would have posed operational risks for its placement and removal. However, an alternative solution was found, as described in the following.

### 3.1. Gamma Detection Mission

**Figure 5:**
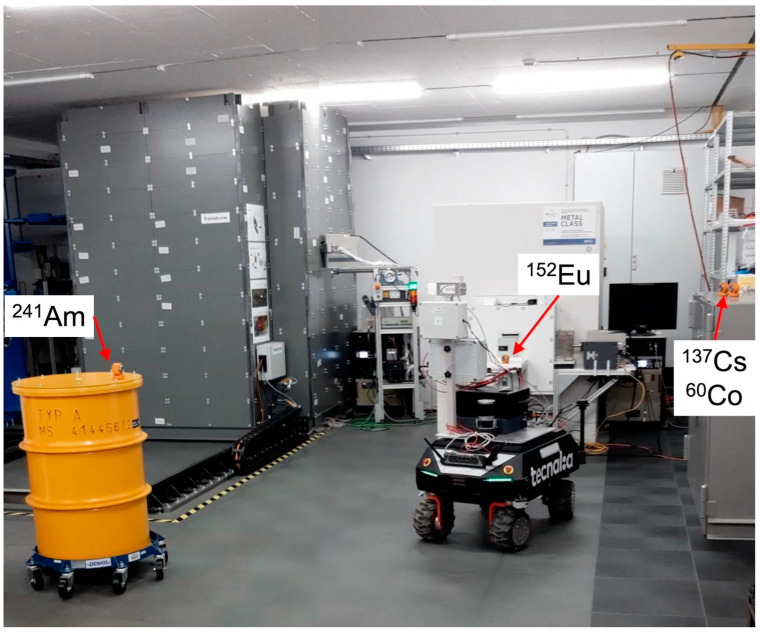
The measurement hall for the gamma mission with the sources: ^241^Am (490 kBq), ^152^Eu (465 kBq), ^137^Cs (282 kBq), ^60^Co (352 kBq).

For the gamma detection mission, four sources were placed in three locations inside the measurement hall (^241^Am (490 kBq), ^152^Eu (465 kBq), ^137^Cs (282 kBq) plus ^60^Co (352 kBq)), as shown in Figure 5. The UGV was sent into the hall and teleguided from a distance of several meters by an operator standing beyond a chain, which delimited the radiation area. Even though the path included continuous motion and pauses, we subdivided it into 53 main steps of 20 s duration. The MiniRadMeter kept acquiring data throughout the mission, alternating measurements of gamma and neutrons every 10 s. 

**Figure 6:**
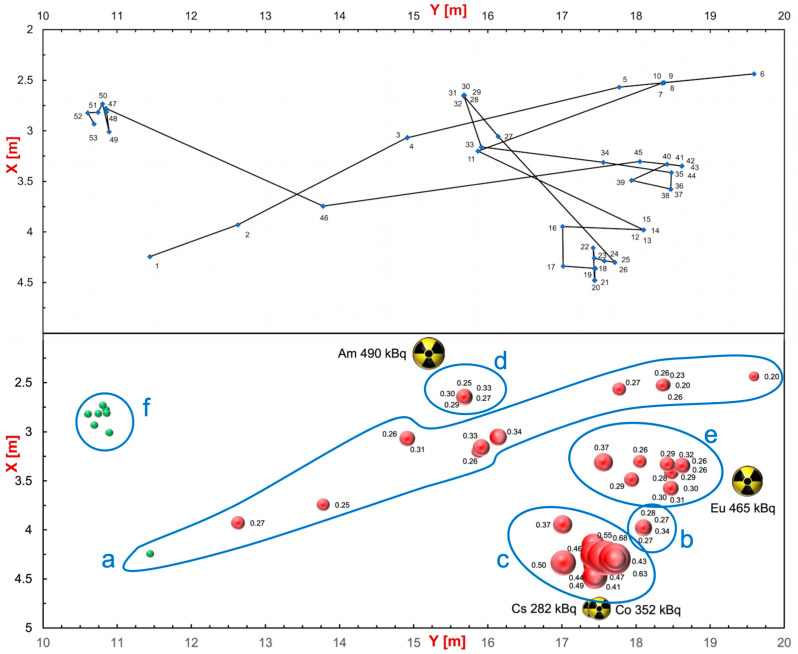
Global summary of the gamma mission. (**Top**) The path followed by the UGV with the indication of the 53 positions. (**Bottom**) Bubble plot with the corresponding measured dose rates with numbers (in µSv/h) and bubble size. The indicated tags **a**–**f** correspond to the regions as listed in Table 1. The position and activity of the sources are also shown.

**Figure 7:**
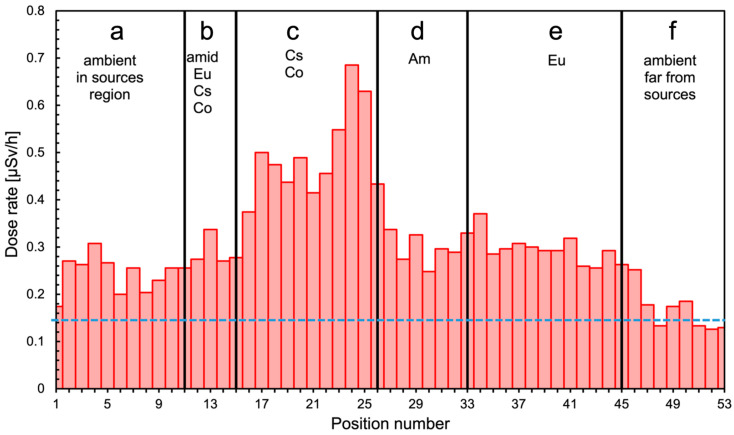
Measured dose rate as a function of the position number. The full path is roughly grouped into six regions denoted by **a**–**f,** as listed in Table 1.

**Figure 8:**
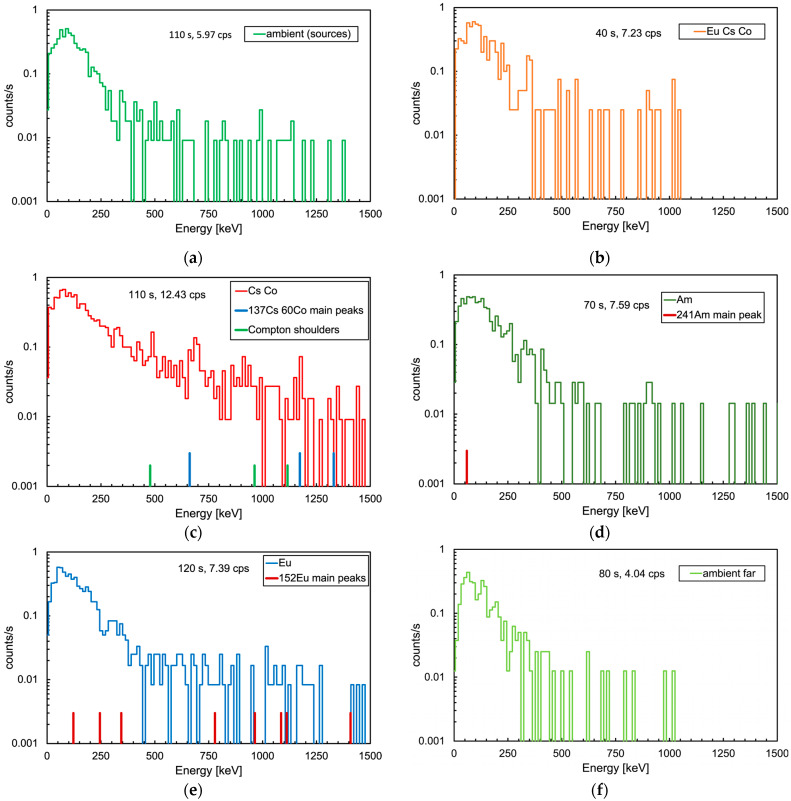
Gamma spectra collected during the mission. (**a**) Ambient, not far from the sources. (**b**) In the region between the ^137^Cs, ^60^Co and ^152^Eu sources. (**c**) In front of the ^137^Cs and ^60^Co sources, the position of the three gamma peak energies and their corresponding Compton shoulders are indicated. (**d**) In front of the ^241^Am source, the position of the gamma peak energy is indicated. (**e**) In front of the ^152^Eu source, the positions of the main gamma peak energies are indicated. (**f**) Ambient, far from the sources.

Figure 6 shows a global summary of the gamma mission. The top plot shows the followed path with the indication of the 53 positions, whereas the bottom bubble plot indicates the corresponding measured dose rates with numbers, in µSv/h, and bubble size. The position and activity of the sources are also shown. The counting rate data in each position were converted into dose rate and plotted as shown in Figure 7 as a function of the position number, providing a useful profile along the UGV inspection path. The full path was roughly grouped into six locations, denoted by **a**–**f** tags in the plot, and the corresponding measured average dose rates are reported in Table 1. In Figure 8, we show the six spectra measured during the mission in the respective locations. Despite the low collected statistics due to the short data collection time, one can appreciate differences in the shape and in the overall counting rates. In particular, in Figure 8c–e, we also show the position of the main peaks expected from the employed sources (in **c** also the position of the Compton shoulders is indicated), which provides a hint to a possible isotope identification along with the measured counting rates indicated in the plots and listed in Table 1. Obviously, in order to (try to) identify the emitting isotopes, one would require the UGV to stop in front of the positions of interest for a longer time. 

### 3.2. Neutron Detection Mission

**Figure 9:**
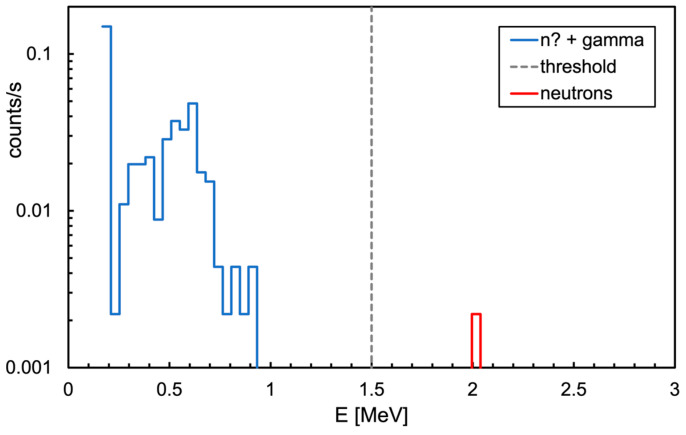
Overall spectrum of the energy deposited in the neutron detector throughout the 530 s of the mission.

**Figure 10:**
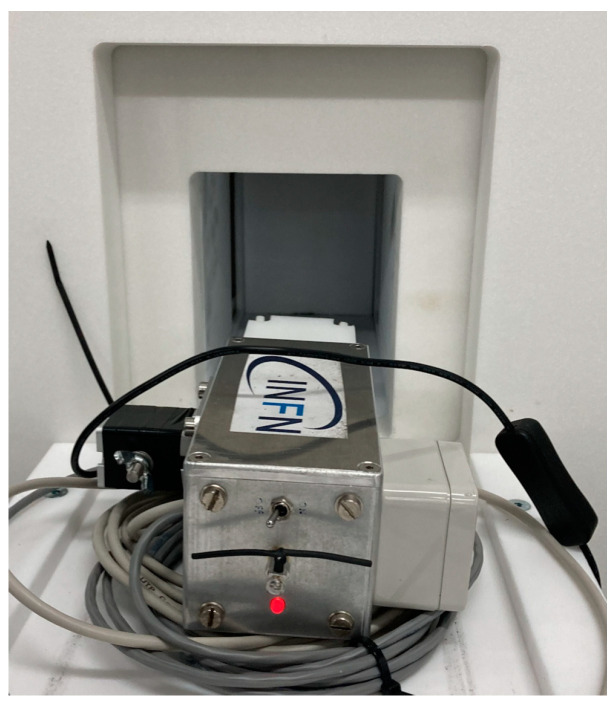
The MiniRadMeter placed on a tray in front of the neutron generator output window.

**Figure 11:**
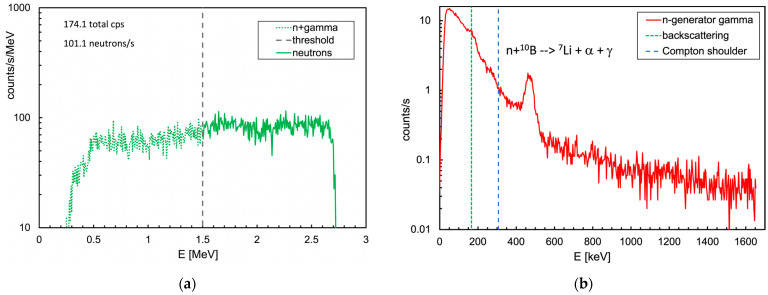
(**a**) Spectrum of the energy deposited in the SiLiF detector. The threshold at 1.5 MeV, recommended for an optimal gamma rejection [15,17], is highlighted. (**b**) Spectrum recorded by the gamma detector while measuring at the neutron generator. The expected peak at 473 keV is visible, along with the bump at 166 keV from its Compton backscattering.

The overall spectrum of the energy deposited in the neutron detector during the gamma mission, shown in Figure 9, basically indicates that there was no neutron emission in the area (one neutron detected at 530 s was compatible with the environmental background). Profiting by the availability of a neutron generator inside the shielded hall at AiNT, a dedicated neutron mission was performed with the MiniRadMeter placed on a tray facing its output window (Figure 10). The generator basically consists of a very compact (≈40 cm long) electrostatic accelerator and can exploit the deuterium–deuterium and the deuterium–tritium collisions (DD and DT in short) with the reactions ^2^H (^2^H, n) ^3^He and ^3^H (^2^H, n) ^4^He. Deuterium is accelerated at several hundred keV energy to hit a deuterated or tritiated solid target, and the outcoming neutron kinetic energies are 2.5 and 14.4 MeV, respectively. As tritium is radioactive, the DD mechanism is more convenient, even though it produces lower energy neutrons. However, for our purposes, DD was more than enough as we were not interested in high energy. The produced neutrons undergo some moderation inside a big box of graphite blocks surrounding the generator, with a flux in the predefined sample irradiation position of the order of ≈5 × 10^4^ n/cm^2^/s. A shield of borated polyethylene surrounds the box to strongly suppress the outcoming neutron flux. In our case, the MiniRadMeter was not placed in the sample position, but rather out of the box in front of a window opening where some graphite blocks had been removed to allow neutrons to come through. The neutron flux and spectrum in such a position and geometrical arrangement were not well known, even though they were estimated between 1 × 10^4^ and 4 × 10^4^ n/cm^2^/s. This is why the mission was prudentially performed with the MiniRadMeter alone in order to prevent possible damage to the UGV and/or other equipment onboard. An acquisition of 150 s was run and a rate of about 100 counts/s of thermalized neutrons was measured. Given the 5% detection efficiency, this corresponded to ≈2000 thermal neutrons/s/cm^2^ on the SiLiF detector. Assuming a ≈10% average thermalization efficiency [2], we roughly estimated an incoming flux of the order of 2 × 10^4^ n/cm^2^/s, compatible with the coarse expectation from the generator in the employed operating conditions. In Figure 11a, we show the deposited energy spectrum where the threshold of 1.5 MeV, recommended for an optimal gamma rejection [15,17], is highlighted. In this particular case, the only gamma rays to be expected were 473 keV and 2.2 MeV, produced by neutron capture on boron and hydrogen, respectively, in the borated polyethylene shield surrounding the generator. The contribution of these gamma rays to the spectrum came only from Compton scattering and, above 1.5 MeV, it was basically null. As a check, we also looked at the spectrum recorded by the gamma detector while measuring at the neutron generator, as shown in Figure 11b. A prominent peak at 473 keV was visible, as expected, along with the bump at 166 keV from its Compton backscattering. The long tail at higher energy was likely due to the Compton scattering of the 2.2 MeV gamma rays and by neutron activation of the surrounding materials.

## 4. Discussion

Since the gamma detector is not simply a counter, but also produces a spectrum, it makes it possible to measure the dose rate in a more appropriate fashion, i.e., by measuring the energy effectively deposited on the crystal. Thus, the average dose rates in the **a**–**f** regions were calculated by means of the weighted integral of each spectrum, according to Equation (1).

(1) $$D=\frac{\sum_{E}E\cdot c\left(E\right)\cdot e}{t\cdot m}\cdot 10^{6}\cdot 3600\;\left[\mu \mathrm{Sv}/h\right]$$

where

*D* = dose rate in µSv/h;

*E* = energy value of the spectrum bin in eV;

*c* = spectrum counts in the bin E;

*e* = 1.6 × 10^−19^ is the electron charge in Coulomb;

*t* = data collection duration in seconds;

*m* = mass of the crystal, i.e., 0.00451 kg;

10^6^ is required to go from Sv to µSv, and 3600 is the number of seconds in 1 hour.

**Figure 12:**
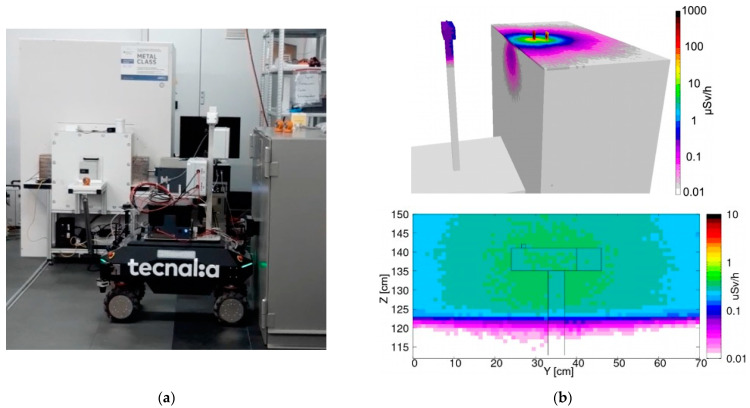
(**a**) The UGV in front of the ^137^Cs + ^60^Co sources. (**b top**) Simulated 3D dose rate profile. (**b bottom**) Dose rate distribution in a vertical plane containing the gamma detector.

**Figure 13:**
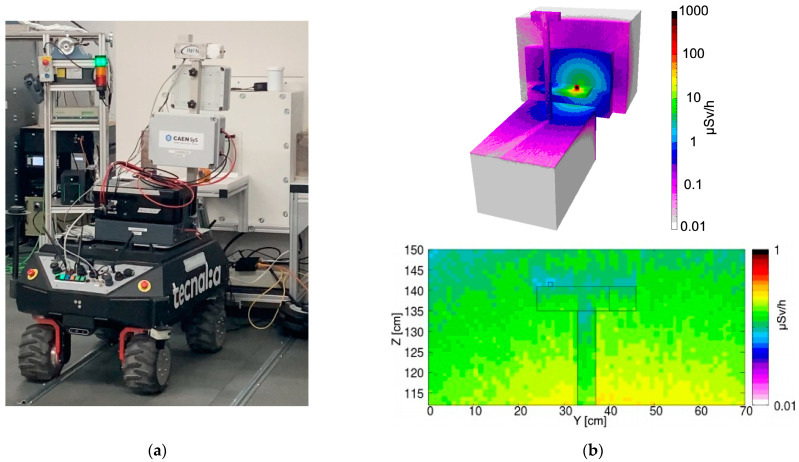
(**a**) The UGV in front of the ^152^Eu source. (**b top**) Simulated 3D dose rate profile. (**b bottom**) Dose rate distribution in a vertical plane containing the gamma detector.

**Figure 14:**
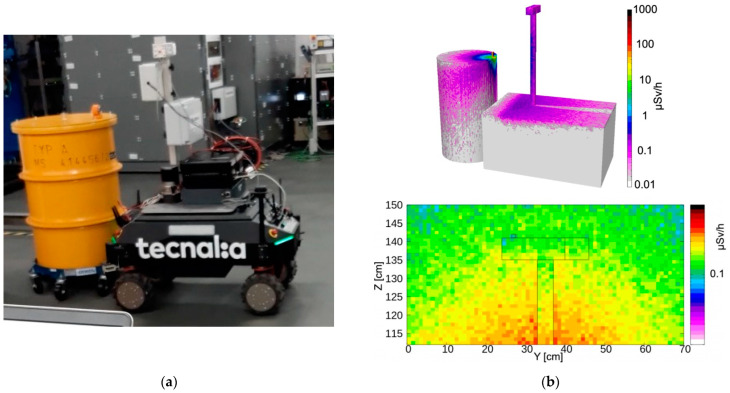
(**a**) The UGV in front of the ^241^Am source. (**b top**) Simulated 3D dose rate profile. (**b bottom**) Dose rate distribution in a vertical plane containing the gamma detector.

The obtained values, along with their statistical uncertainty, are listed in Table 1 and denoted as measured. The agreement between direct and measured values looked fairly reasonable. However, as a further verification of the correctness of the order of magnitude, we also ran a few simulations using FLUKA [27] and Flair [28] software. A skeletal geometric reconstruction of the UGV in front of the sources was reproduced and the approximate distances between the sources and the gamma detector were estimated by means of several videos and photographs taken during the mission. Three configurations were simulated, and the resulting expected dose rate profile was produced for the three main positions in front of the ^137^Cs + ^60^Co (Figure 12), ^241^Am (Figure 13) and ^152^Eu (Figure 14) sources, along with the corresponding pictures. Even though the exact geometry was not implemented, the resulting simulated dose rate values, listed in Table 1, were fairly close to the values obtained with the MiniRadMeter.

**Figure 15:**
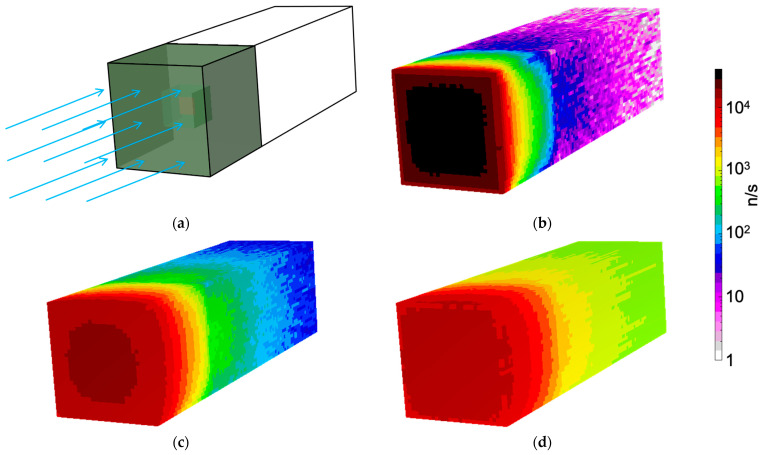
Neutron flux distribution in the MiniRadMeter, simulated with an initial monoenergetic beam of 2 × 10^4^ neutrons/cm^2^/s impinging on the moderator. (**a**) Simulated geometry. (**b**) Beam energy 25 meV. (**c**) Beam energy 100 keV. (**d**) Beam energy 1 MeV.

A simulation was also performed for the neutron measurement with the generator. The neutron spectrum in the measurement position was not known, as it was outside the generator itself. We presumed the real spectrum should have a well thermalized component, because of the massive graphite and polyethylene, and a partially moderated higher energy one exiting the access window almost directly. The proportions were not known; therefore, we simulated separately three beams of 2 × 10^4^ n/cm^2^/s, over a 6 × 6 cm^2^ area, for the three impinging neutron energies 25 meV, 100 keV and 1 MeV. The resulting flux distributions are shown in Figure 15.

**Figure 16:**
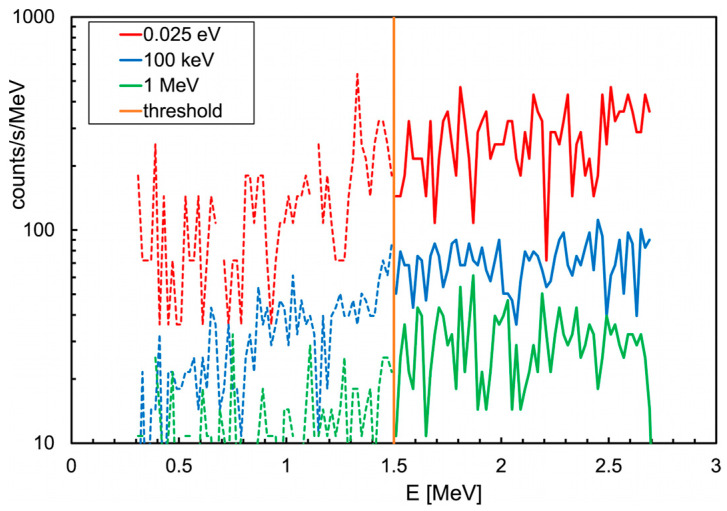
The deposited energy spectra in the silicon detector for the three simulated neutron fluxes of Table 2 and Figure 15b–d.

By looking at Figure 15b, one can see how the thermal neutron flux was mainly localized in the moderator, where neutrons were kept bouncing for a while and then scattered away or captured in hydrogen, while a small fraction interacted with the ^6^LiF converter and the produced ^3^H and/or ^4^He were detected by the silicon diode. When the neutron beam energy was increased to 100 keV (Figure 15c) and to 1 MeV (Figure 15d), the flux increased more deeply in the moderator and in the electronics box. The three corresponding spectra of the energy deposited in the silicon detector, reported in Figure 16, showed that the assumptions about the real flux were reasonable by comparison with Figure 11a. The total and the neutron counts (i.e., for E > 1.5 MeV) in the spectra of Figure 11 and Figure 16 are listed in Table 2. 

Two important features of the MiniRadMeter to be investigated were the minimum detectable activity (MDA) and the minimum detectable neutron flux (MDNF) for the gamma and for the neutron detectors, respectively. To calculate the MDA in the described operating conditions, we referred to the 662 keV gamma rays from a point-like ^137^Cs source, knowing that the gamma interaction probability in 1 cm of CsI(Tl) was about 0.3 [26] and the geometrical efficiency was a function of the distance between the source and the detector. Considering a data collection duration of 10 s, and an average background rate of about 4.04 counts/s measured in 80 s as reported in Figure 8f and Table 1, the MDA_95%_, i.e., at 95% confidence level, could be calculated (in Becquerel) by means of Equation (2) [29,30,31]

(2) $$MDA_{95\%}=\frac{3+3.29\cdot \sqrt{R_{b}\cdot t_{s}\cdot \left(1+\frac{t_{s}}{t_{b}}\right)}}{t_{s}\cdot \varepsilon }$$

where

*R_b_* = background count rate;

*t_s_* = sample count time;

*t_b_* = background count time;

*ε* = detector efficiency in counts/decay, i.e., 0.3 times the geometric efficiency.

**Figure 17:**
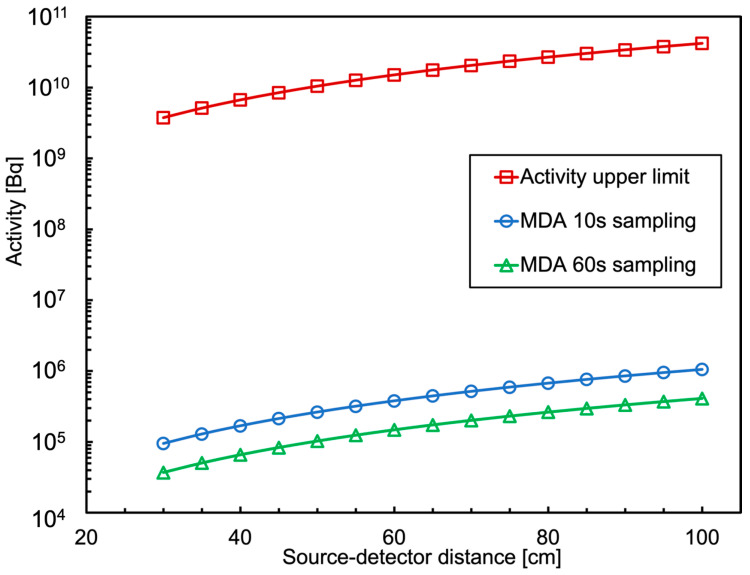
Operational range of the MiniRadMeter gamma detector, between minimum detectable activity and activity upper limit, as a function of its distance from a point-like ^137^Cs source. Shown are two MDA cases with 10 s and 60 s sampling times.

The results as a function of the source-detector distance are plotted in Figure 17, considering that the position where the MiniRadMeter was installed on the UGV was such that the minimum distance from a possible radiation source was about 30 cm. Just for comparison, the same calculation was performed and the results plotted for another case, where we assumed a sampling time of 60 s and a background collection time of 300 s. The plot can be taken as a reference, as it shows that even a scan of 10 s makes it possible to successfully detect a gamma source with activity of the order of a few 10^5^ Bq within one meter distance. The upper limit of the system is the high-rate capability. Indeed, the 20 µs signal integration time we employed can be lowered to 5 µs with little influence on the energy resolution, thus implying a counting rate limit of the order of 100,000 counts/s. By means of the previous calculation, we translated this limit into an activity upper limit as a function of the distance, which is also plotted in Figure 17. 

A slightly modified version of Equation (2), where the efficiency *ε* is replaced by the average intrinsic detection efficiency, can be exploited to calculate the MDNF. The measurement during the gamma mission produced only one neutron count in 530 s. For our estimate, we assumed this as a background rate and considered a sampling duration of 10 s. In the case of a thermal neutron flux, therefore running without moderator, the detection efficiency was 5% and gave rise to MDNF ≈ 6.9 neutrons/cm^2^/s. If running with the moderator, thus assuming a worse average detection efficiency *ε* ≈ 0.5% for neutrons between thermal and 1 MeV energy, the resulting value was MDNF ≈ 69 neutrons/cm^2^/s.

**Figure 18:**
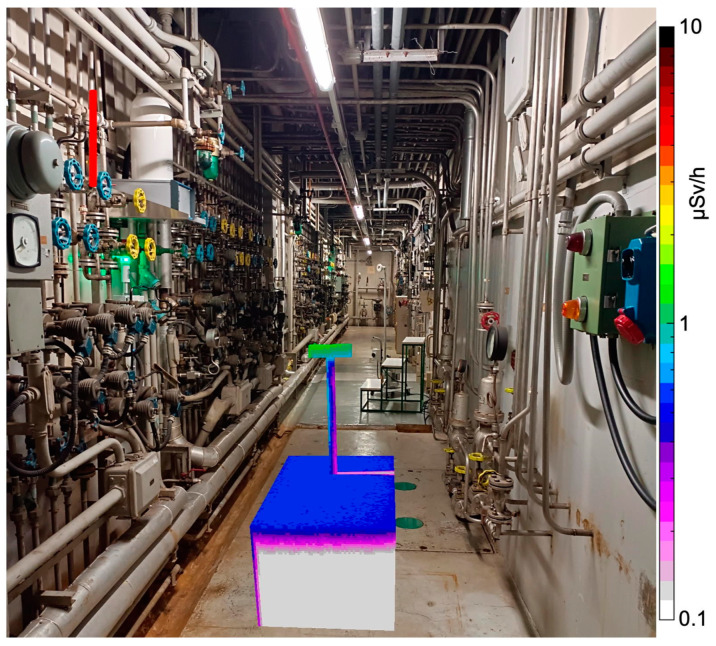
Simulation of a hypothetical environment to be inspected: a long tunnel with pipes, valves and cables. A 70 cm vertical pipe section, highlighted in red for clarity (top left), is assumed to be contaminated with 10 MBq of ^60^Co. The dose rate scale only refers to the UGV.

**Figure 19:**
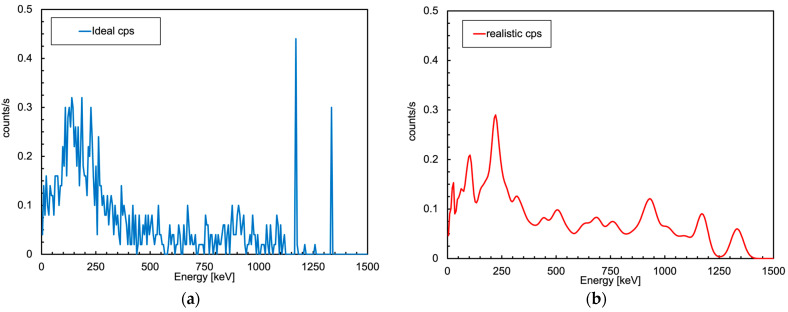
Simulated spectrum of the energy deposited into the gamma detector in the configuration of Figure 18 in 10 s acquisitions. (**a**) Ideal case with perfect energy resolution. (**b**) Realistic case convoluted with the typical detector resolution.

In the end, after validating the reliability of the numerical simulations and calculating the detection limits of the MiniRadMeter, we set up a simplified geometry in order to see what the detector performance could be in a realistic condition. In Figure 18, we show a presumable environment to be inspected—a long tunnel with pipes, valves and cables. We assumed that a 70 cm section of vertical pipe, whose midpoint was located at 2.5 m height, was contaminated with 10 MBq of ^60^Co. While the UGV moved along the corridor, it reached a position where the distance between the pipe’s midpoint and the gamma detector was about 2 m. The calculated MDA for such a distance in ten seconds was ≈4 MBq for ^137^Cs, which roughly scaled down to 2 MBq for ^60^Co due to the two gamma rays emitted per decay, meaning that the activated pipe would be easily detected. The simulated counting rate was 20 counts/s. The deposited energy spectrum (ideal) and the corresponding one convoluted with the typical detector resolution of 7% FWHM at 662 keV are shown in Figure 19.

## 5. Conclusions

The MiniRadMeter device, developed in the framework of the H2020 CLEANDEM project, was installed on an unmanned ground vehicle. A simulated field mission was performed in a shielded hall with four “hidden” radioactive sources and a neutron generator to prove its capability as a quick scanner for gamma and neutron radiation mapping in case of decommissioning and/or nuclear accident remediation. The data collected during the mission were validated by numerical simulations. Despite its low cost and compact size, the MiniRadMeter showed an interesting performance in terms of reliability and sensitivity. The quantified minimum detectable gamma activity and neutron flux make it suitable for the task it was aimed for. The overall features of this device make it an interesting candidate for installation onboard small flying drones to be operated indoor or outdoor, in environments where it can fly in hummingbird fashion for close inspection of possibly contaminated rooms and locations.

## Figures and Tables

**Table 1:** Results from measurements and simulations in the six areas investigated. The direct dose rate was obtained by multiplying the measured count rate by the dose conversion factor of 0.037 (µSv/h)/(counts/s). For the measured and simulated dose rates, see the following chapter. The quoted uncertainties are statistical only.

Location	Tag	Run Time [s]	Count Rate [cps]	Average Dose Rate (Direct) [µSv/h]	Average Dose Rate (Measured) [µSv/h]	Dose Rate (Simulation) [µSv/h]
ambient, in sources region	a	110	5.97	0.22 ± 0.009	0.13 ± 0.005	-
amid ^152^Eu, ^137^Cs, ^60^Co	b	40	7.23	0.27 ± 0.016	0.17 ± 0.010	-
^137^Cs, ^60^Co	c	110	12.43	0.46 ± 0.012	0.44 ± 0.012	≈0.4
^241^Am	d	70	7.59	0.28 ± 0.012	0.18 ± 0.008	≈0.12
^152^Eu	e	120	7.39	0.27 ± 0.009	0.18 ± 0.006	≈0.1
ambient, far from sources	f	80	4.04	0.15 ± 0.008	0.07 ± 0.004	-

**Table 2:** Total and neutron counts (i.e., for E_dep_ > 1.5 MeV) in the spectra of Figure 11 and Figure 16.

Flux	Total Counts/s	Neutrons/s	Note
2 × 10^4^ n/cm^2^/s, 25 meV	487	330	simulation
2 × 10^4^ n/cm^2^/s, 100 keV	125	86	simulation
2 × 10^4^ n/cm^2^/s, 1 MeV	50	36	simulation
measured	174	101	experimental

## Data Availability

The data presented in this study are available on request from the corresponding author.
